# The conduct of maternal and perinatal death reviews in Oyam District, Uganda: a descriptive cross-sectional study

**DOI:** 10.1186/s12905-016-0315-5

**Published:** 2016-07-14

**Authors:** Caroline Agaro, Jolly Beyeza-Kashesya, Peter Waiswa, Juliet N. Sekandi, Suzan Tusiime, Ronald Anguzu, Elizabeth Ekirapa Kiracho

**Affiliations:** Makerere University School of Public Health, Kampala, Uganda; Depertment of Health Policy, Planning and Management Makerere University School of Public Health, Kampala, Uganda; Department of Obstetrics and Gynaecology, Makerere University, College of Health Sciences, Kampala, Uganda; Department of Epidemiology & Biostatistics Makerere University School of Public Health, Kampala, Uganda

**Keywords:** Maternal and perinatal deaths, Maternal Perinatal Death Review

## Abstract

**Background:**

Uganda like many developing countries still experiences high levels of maternal and perinatal deaths despite a decade of maternal and perinatal death review (MPDR) program. Oyam district has been implementing MPDR since 2008 with varying successes among the health facilities. This paper presents the factors that influence the conduct of maternal and perinatal death reviews in Oyam District, Uganda.

**Methods:**

This was a cross-sectional study where both qualitative and quantitative data were collected. Semi-structured interviews were administered to 66 health workers and ten key informants (KIs) to assess the factors influencing the conduct of MPDR. Univariate and Bivariate analysis of quantitative data was done using SPSS version 17.0. A Pearson Chi-Square test was done to determine factors associated with conduct of MPDR. Factors with a *p-*value < 0.05 were considered statistically significant. Qualitative data was analyzed using content analysis.

**Results:**

Only 34.8 % of the health workers had ever participated in MPDR. The factors that influenced conduct of MPDR were existence of MPDR committees (*p <* 0.001), attendance of review meetings *(p* < 0.001) and knowledge of objectives of MPDR *(p < 0.001)*, implementation of MPDR recommendations (*p < 0.001)*, observed improvement in maternal and newborn care (*p < 0.001)* and provision of feedback *(p < 0.001)*. Hindrance to conduct of MPDR was obtained from KIs: the health workers were not made aware of the MPDR process, committee formation and training of MPDR committee members was not effectively done, inadequate support supervision, and lack of financial motivation of MPDR committee members. Challenges to MPDR included: heavy workload to health workers, high number of perinatal deaths, and non-implementation of recommendations.

**Conclusion:**

The proportion of maternal and perinatal death reviews conducted in Oyam was low. This was due to poor initiation of the review process and a lack of support supervision. The district and Ministry of Health needs to put more emphasis on monitoring the conduct of maternal and perinatal death reviews by: forming and training MPDR committees and ensuring they are financially supported, providing overall coordination, and ensuring effective support supervision.

**Electronic supplementary material:**

The online version of this article (doi:10.1186/s12905-016-0315-5) contains supplementary material, which is available to authorized users.

## Background

Women continue to die from preventable complications of pregnancy and childbirth. The major killers include postpartum haemorrhage, sepsis, severe pre-eclampsia/eclampsia, abortion complications and obstructed labour. This is attributed to poor quality of care associated with absence of skilled health personnel during pregnancy and childbirth, the lack of emergency obstetric and newborn care services to deal with the complications, and ineffective referral systems [1, 2]. The newborns mainly die due to birth asphyxia, sepsis, and prematurity. Studies reported that poor foetal heart monitoring during labour was indirectly associated with over 40 % of perinatal death, and health workers’ knowledge of newborn care was only 31 % [3–5].

Uganda has high maternal mortality ratio of 438/100,000 live births, 435/100,000 live births in 2006 and 524/100,000 live births in 2000. A perinatal mortality rate of 27/1000 pregnancies in 2011, 46/1000 pregnancies in 2006 and 55/1000 pregnancies in 2000 [6, 7]. In efforts to achieve millennium development goals (MDGs) 4 and 5, the ministry of health (MoH), Uganda tried to put in place mechanisms to improve maternal and newborn health using maternal death reviews as a quality improvement tool. In 2008, maternal deaths were made a notifiable event. In addition, perinatal death reviewing was incorporated because of its close linkage with maternal deaths. A National Committee on Maternal Perinatal Death Review (NCMPDR) was established in 2008 and it oversees the review process and makes strategies for implementation of recommendations. A 5 year maternal, perinatal and child death review strategic plan 2010–2015 and MPDR guidelines have been developed [8]. In addition, MPDR is considered an important monitoring tool in the Uganda National Roadmap for the accelerated reduction of maternal and newborn mortality.

Whereas maternal and perinatal death reviews have been adopted as a key strategy to build capacity and inculcate a culture of continuous quality improvement, maternal and perinatal death review activities are not implemented widely. By 2011, only 20 districts in Uganda were reporting maternal deaths [9]. Furthermore, there are very few national/regional mentors and supervisors to support districts [9]. Some health facilities have formed MPDR committees though most of them are not functional or are not reporting. The conduct of MPDR is irregular yet it is a presidential directive. Revitalization of MPDR was done by setting up and training MPDR health facility (HF) committees and health workers in pilot hospitals, this has continued to be rolled out to health facilities [10].

In 2008, United Nations Population Fund, formerly the United Nations Fund for Population Activities (UNFPA) supported Oyam district to initiate MPDR by facilitating the formation and training of MPDR committees at health facilities, health sub-district and district levels. However the success in conduct of MPDR in Oyam district has not been evaluated. Little is known about the current MPDR status, which health units are reviewing and what could support and/or hamper MPDR. In addition, little is known about which system level barriers exist and need to be addressed to ensure MPDR accomplishes its goal. This paper reports results of assessment of the MPDR process in Oyam district. It critically looks at the conduct of MPDR among the different health facilities, focusing on the review process and challenges and lessons learned and the suggested solutions to address challenges.

## Methods

### Study setting

The study was conducted between March and May 2012 in Oyam district. Oyam is located in Mid-Northern Uganda and is bordered by Gulu district in the North, Pader in North East, Lira and Kole in the East, Apac in the South, Kiryadongo and Nwoya in the West. It has a population of 366,200 projected from the 2002 population census. Oyam is one of the districts that were ravaged by the lord’s resistance army (LRA); an insurgency that affected the Northern Uganda region for over 20 years, disrupting health systems. The district health structure is comprised of 24 health facilities that include one hospital, one HC IV (min-hospital with operating theatre, which provides comprehensive emergency obstetric care), 5 HC III’s (conducts deliveries but refers those mothers with complications and those that need operation or advanced care) and 17 HC II’s (very basic unit that provide antenatal care but is not mandated to conduct deliveries though some do). The actual maternal mortality ratio of Oyam district is not known but data from the district health office puts it at 309/100,000 live births and perinatal mortality rate of 43.8/1000 live births, with the proportion of health facility deliveries estimated at 38.9 % [10, 11].

### Study design, population and sampling

This was a cross-sectional study with both qualitative and quantitative methods of data collection. The study population comprised of health facilities, health workers working in maternity and children’s wards, district health teams, in-charges of health facilities and chairpersons of maternal and perinatal deaths review committees. All the health facilities (two) that are expected to conduct MPDR as mandated by policy were selected; these included one hospital, 1 health centre (HC) IV. In addition, we selected all the 5 HC III’s because the UNFPA program had extended MPDR training to them. All the health facility maternal and perinatal deaths that occurred from 2008 to 2011 in the sampled health facilities were reviewed. In addition, 10 KIs including four district health team members, 4 health facility in-charges and two chairpersons of maternal and perinatal death review committee who were specifically selected based on their role in MPDR process right from policy to review recommendations and actions formulation. Sixty-six health workers working in maternity and children’s wards were also purposively selected for the study because they participate in MPDR and are conversant with the review process.

### Data collection and management

The main outcome variable was conduct of maternal and perinatal death review which was measured by the proportion of maternal and perinatal deaths reviewed calculated from all health facility maternal and perinatal deaths that occurred from 2008 to 2011. Other variables included: existence and functionality of maternal death and perinatal review committees, frequency of review meetings, recommendations and actions taken upon maternal and perinatal death review recommendations, participation of senior staff in the MPDR meetings, support supervision and follow-up on MPDR activities, number of health workers in health facilities, health workers knowledge of MPDR, attitudes of health workers towards MPDR, MPDR practices, perceptions of health workers about MPDR, motivation to conduct MPDR, and training on MPDR.

Data was collected by trained research assistants using pre-tested tools.

A checklist was used to abstract data on deliveries, maternal and perinatal deaths from the integrated maternity register*.* A KI guide was used for KI interviews with the district health team members, in-charges of health facilities and chairpersons of the MPDR committees*.* Information explored was based on the variables from the quantitative data (Additional file 1). They included initiation of MPDR in the district, the role of the district in the process, existence and structure of review committees, and process of reviews, actions taken on the review recommendations and dissemination of findings. The challenges faced by the review process and how they can be improved. The interview guide covered components of an ideal review model to assess composition, timing and frequency of review meetings, analysis of results, making the recommendations, and use of recommendations to make action plans. Interviews were conducted in English and lasted about 40–45 min. Interviews were audio recorded with consent of the respondent, translated, and transcribed by the research assistants that participated in the interviews.

A Semi-structured questionnaire was used to generate data from health workers in the maternity and children’s wards on service delivery and health workforce factors that influence the conduct of MPDR. These included their level of awareness, attitude, practices and perceptions towards MPDR. In both qualitative and quantitative interviews the study explored the level of implementation of review recommendations and interviewees’ suggestions for improvement of their health facilities.

### Data management and analysis

The Quantitative data collected using semi-structured questionnaires and the checklist were pre-coded and entered into the computer using Epi-Data version 3.1 software. Data was cleaned and stored on a daily basis. Data was exported to and analyzed using SPSS statistics 17.0 software. Univariate analysis was done to describe study variables using frequency tables. The output was presented in tables, frequencies and proportions. Bivariate analysis was done using Pearson Chi-Square test to assess factors associated with conduct of MPDR.

The qualitative audio-recorded raw data was transcribed and read through together with field notes to see whether the content had complete responses, and also to remove the phrases with vague meanings. All interviews and field notes were read through many times to become very familiar with the data, and identify patterns in the data, and coded using a content analysis approach. Coding was led by [CA] the first author assisted by [JBK] the second author, together they identified major themes and sub-themes as they emerged within the data. The coding scheme was reviewed and approved by all authors. The major themes were determined a priori by the main outcomes from the quantitative analysis. Responses were enumerated by question and objective and banded into [*Few (1–3) respondents, ** majority (4–6) respondents, *** Most (7–10) respondents]. In this paper, we present the qualitative data that complement the quantitative data to highlight the factors that influence the conduct of MPDR, the challenges of service delivery and health work force to conduct MPDR and what respondents suggested as solutions to address these challenges. We have selected quotations that illuminate the themes and are presented verbatim.

## Results

Sixty-six (66) health workers working in the maternity, children’s wards and outpatient departments during the period of study were interviewed. Ten (10) KI interviews were conducted with 4 district health team members, four in-charges of health facilities and two chairpersons of MPDR committees.

This table shows health facilities in which the study was conducted by name, level, location and ownership of the health facility and the number of health workers who participated in the study from each health facility. Out of the seven health facilities, three were private not for profit; (One hospital and 2 HC III’s). The hospital had the highest proportion of health workers participating in the study (Table 1).

From 2008 to 2011, there were 18,909 institutional deliveries in the health facilities studied. A total of 827 deaths were registered at the health units (68 maternal & 759 perinatal deaths) with Aber hospital registering the highest maternal (46) and perinatal (479) deaths. More maternal 48/68 (71 %) than perinatal deaths 253/759 (33.3 %) were reviewed. The audits were only conducted in Aber Hospital 53.6 % and Anyeke HC IV 11.1 %. While four of the seven (4/7) health facilities had MPDR committees, only two MPDR committees were functional (Table 2).

Avoidable factors identified included patient factors such as late referrals, health system issues and the lack of training of MPDR committees. The actions taken were appropriate and according to the recommendations, which came from the avoidable factors. Majority of cases had the late referrals as the single main avoidable factor (Table 3).

### Factors that influence conduct of MPDR in Oyam district

Service delivery factors and health workforce factors influenced the conduct of MPDR in Oyam district. Among the service delivery factors was the existence and functionality of maternal and perinatal review committees. Within the health workforce factors, awareness, attitudes and perceptions of health workers on the previous conduct of and usefulness of MPDR results were important for the participation in MPDR. In addition, service delivery and health work force challenges to the conduct of MPDR, and the proposed solutions to address the challenges influence conduct of MPDR.

Table 4 shows the Univariate and bivariate analysis of the service delivery factors that influence conduct of MPDR by health workers interviewed. At univariate analysis, 65.2 % of respondents were from maternity wards while 15.2 % were from the children’s wards. Majority, 63.6 % of the health workers said they had MDPR committees in their health facility, though only 34.8 % (23/66) reported to have ever participated in an MPDR meeting. Almost half (48.5 %) of them said the core MPDR members and senior members of their health facilities attend review meetings. In this study, a third (31.8 %) of the respondents knew at least two recommendations of the maternal and perinatal death review committee in their health facilities. Only 39.4 % knew actions that had been implemented following the MPDR recommendations. Less than half (48.5 %) of the respondents noticed improvement, as a result of MPDR, in maternal and newborn care provided.

**Table 1:** Health facilities and corresponding number of health workers interviewed

Location	Name of HF^a^	Level of HF	Ownership of HF	No. of HWs^b^ *N* = 66 (%)
Oyam South HSD	Aber	Hospital	NGO(PNFP)	24/66 (36.4)
	Agulurude	HC III	Government	8/66 (12.2)
	Minakulu	HC III	NGO(PNFP)	7/66 (10.6)
Oyam North HSD	Anyeke	HC IV	Government	9/66 (13.6)
	Iceme	HC III	NGO(PNFP)	5/66 (7.6)
	Ngai	HC III	Government	6/66 (9.1)
	Otwal	HC III	Government	7/66 (10.6)

^a^ *Health facility*

^b^ *Health workers*

**Table 2:** Proportion of maternal and perinatal deaths audited between 2008 and 2011

Health facility	Deliveries	Maternal deaths		Perinatal deaths	
		Deaths	Reviewed (%)	Deaths	Reviewed (%)
Aber Hospital	6498	64	47 (73.4)	479	244 (50.9)
Agulurude HC III	2075	0	0	45	0
Anyeke HC IV	3126	2	1(50)	88	9 (10.2)
Iceme HC III	1277	1	0	9	0
Minakulu HC III	1474	0	0	39	0
Ngai HC III	1921	0	0	6	0
Otwal HC III	2538	1	0	93	0
Total	18,909	68	48 (71)	759	253 (33.3)

**Table 3:** Avoidable factors identified and actions taken following MPDA recommendations

Avoidable factors identified			
Avoidable factors identified	No. of cases	Actions taken following recommendations	No. of cases
Late referrals	***	Early referrals	***
Late requisition of drugs and supplies	*	Early requisition of drugs and supplies	*
Non-use or improper use of Partographs	*	Strict use of Partographs	*
Lack of training on MPDR	*	Training of health workers on how to use partographs	*
Limited number of staff	*	Continuous medical education on data management	*
		Integrated MPDA support supervision to other activities	*
		Community sensitization	*
		Health education/talks at OPD, ANC	**
		Blood pressure machines procured	*
		Internal transfer of Midwives & Nurses	**
		Follow-up to health facilities	*

Data sourced from field findings from records of MPDR reviews; * Few, **majority, ***Most

**Table 4:** Service delivery factors that influence conduct of MPDR among health workers in Oyam district

Independent Variables	Total No. of participants 66 (%)	No participation *n* = 43 (%)	Participated *n* = 23(%)	χ^2^ (*p-*value)
Cadre of health worker				
Clinician	11 (16.7)	5 (11.6)	6 (26.1)	
Midwife/Nurse	41 (41)	26 (60.4)	15 (65.2)	
Others	14 (21.2)	12 (27.9)	2 (8.7)	4.9(0.179)
Section of health facility where the health provider works				
Maternity	43 (65.2)	23 (53.5)	20 (87.0)	
Children’s ward	10 (15.2)	8 (18.6)	2 (8.7)	
OPD	13 (19.7)	12 (27.9)	1 (4.3)	7.8(0.021)
Knowledge of having an MPDR committee in health facility				
Yes	42 (63.6)	20 (46.5)	22 (95.7)	
No	24 (34.4)	23 (53.5)	1 (4.3)	15.6 (<0.001)
Knowledge of availability of core MPDR committee members				
Yes	32 (48.5)	13 (30.2)	19 (82.6)	
No	34	20 (69.8)	4 (17.4)	16.5 (<0.001)
Senior staff members attend the MPDR meeting				
Yes	32 (48.5)	12 (27.9)	20 (87.0)	
No	34 (51.5)	31 (72.1)	3 (13.0)	20.9 (<0.001)
Knowledge of two MPDR recommendations				
Yes	21 (31.8)	5 (11.6)	16 (69.6)	
No	45 (68.2)	38 (88.4)	7 (21.7)	23.2 (<0.001)
Knowledge of implementation of MPDR recommendations in the HF				
Yes	26 (39.4)	8 (18.6)	18 (78.3)	
No	40 (60.6)	35 (81.4)	5 (13.0)	22.3 (<0.001)
Noticed improvement in maternal and newborn care				
Yes	32 (48.5)	12 (27.9)	20 (87.0)	
No	34 (51.5)	31 (72.1)	3 (13.0)	20.9 (<0.001)

*P value < 0.05 is significant*

### Bivariate analysis of service delivery factors associated with conduct of MPDR

Participation in MPDR meetings was used as a proxy measure of conduct of MPDR. Table 4 shows bivariate analysis results for the association between service delivery factors and conduct of MPDR. Presence of MPDR committee and availability of core MPDR committee members were significantly associated with conduct of MPDR. Most, 96 % of the health workers who had ever participated in MPDR conduct had had MPDR committee available at their health facilities compared to 47 % who had never participated in MPDR meeting (*p* < 0.001). The availability of core MPDR team members was also significantly higher (83 %) among health workers who had ever participated in MPDR meeting compared to 30 % that had never participated in MPDR meeting (*p* < 0.001). There was a statistically significant difference in participation of health workers in MPDR and the communication of recommendation to staff (*p* < 0.001).

The level of awareness about the maternal and perinatal death review was high. Most (81.8 %) of the participants reported that they had ever heard about MPDR. However less than half could remember how MPDR was introduced in their health facilities. When asked about the main objective of MPDR majority (60.6 %) knew the main objective though less than half (37.9 %) said the objectives were not communicated to all the health workers. Moreover, most (78.8 %) of the respondents were not trained on how to conduct maternal and perinatal death review. When asked about their attitude on conducting maternal and perinatal death reviews almost all respondents said they felt encouraged to conduct MPDR. The health workers were also asked about their perceptions on MPDR. Majority, 77.3 % did not agree that conducting MPDR inconvenienced them, and more than half disagreed that conducting MPDR increased their workload. However, 60.6 % reported that the district or ministry of health had not built their capacity to conduct MPDR. Nonetheless, 89.4 % agreed that conducting maternal and perinatal death reviews could improve maternal and perinatal care. See Table 5.

**Table 5:** Health work force factors that influence conduct of MPDR among health workers in Oyam district

Independent Variables	Total No. of participants 66 (%)	No participation *n* = 43 (%)	Participated *n* = 23(%)	χ^2^ (*p-*value)
Awareness				
Health worker knows the main objectives of MPDR				
Yes	40 (60.6)	20(46.5)	20(87.0)	
No	26 (39.4)	23(53.5)	3(−13.0)	10.3 (0.001)
MPDR objectives communicated to health workers				
Yes	25 (37.9)	11(25.6)	14(60.9)	
No	28 (42.4)	24(55.8)	4(17.4)	
Don’t know	13 (19.7)	8(18.6)	5(21.7)	10.2 (0.006)
Health worker trained to conduct MPDR				
Yes	14 (21.2)	11(25.6)	3(13.0)	
No	52 (78.8)	32(74.4)	20(87.0)	1.4 (0.346)
Attitudes				
Health worker feels encouraged to conduct MPDR				
Yes	62 (93.9)	41 (95.3)	21 (91.3)	
No	4 (6.1)	2 (4.7)	2 (8.7)	0.4 (0.435)
MPDR affects how you provide maternal and newborn care				
Yes	62 (93.9)	40 (93.0)	22 (95.7)	
No	4 (6.1)	3 (7.0)	1 (4.3)	0.2 (1.000)
Perceptions				
Conducting MPDR inconveniences you				
Disagree	51 (77.3)	34(79.1)	17(73.9)	
Agree	15 (22.7)	9(20.9)	6(26.1)	0.2 (0.634)
Conducting MPDR increases your workload				
Disagree	37 (56.1)	24(55.8)	13(56.5)	
Agree	29 (43.9)	19(44.2)	10(43.5)	0.0 (0.956)
MPDR improves maternal and newborn care				
Disagree	7 (10.6)	7(16.3)	0(0.0)	
Agree	59 (89.4)	36(83.7)	23(100.0)	4.2 (0.041)
Your capacity built by district or ministry of health to conduct MPDR				
Disagree	40 (60.6)	26(60.5)	14(60.6)	
Agree	26 (39.4)	17(39.5)	9(39.1)	0.0 (0.974)

*P value < 0.05 is significant*

**Table 6:** Functionality and the perceived roles of MPDR committee members

• Community sensitization on benefits of maternal health services to avoid seeking care late
• Creating awareness of other health workers on MPDR
• Review causes and circumstances that lead to maternal and perinatal deaths
• Implementation of suggested recommendations to improve maternal and newborn care
• Follow-up of cases and dissemination of the review findings
• Writing reports and submission of the MPDR reports to the district and ministry of health

The most mentioned responsibility was to review causes and circumstances that lead to maternal and perinatal deaths as sighted by a member of the MPDR committee in the quote “*Every month we sit together to review the causes of maternal and perinatal deaths right from the community up to the time of her death and all the services offered to her”.* ___KI 5 (In-charge HF)

### Bivariate analysis of Health workforce factors associated with conduct of MPDR

Health workforce associated factors included: awareness, attitudes and perceptions of the maternal and perinatal death review process.

### Awareness

About 72 % of health workers who had never participated in MPDR meeting had heard about MPDR. Health workers that had conducted MPDR were aware of the main objectives of MPDR compared to those who had never participated in MPDR meeting (87 % and 47 % respectively; (*p* = 0.001)). In addition, the proportion of health workers at the health unit who had ever participated in MPDR meeting were significantly higher than those who had never participated (16 %) in MPDR (48 % and 16 % respectively; (*p* = 0.001)). However, being trained to conduct MPDR was not significantly associated with participation in MPDR meetings Table 5.

### Attitudes, perceptions of health workers and conduct of MPDR

Conducting MPDR was not different among health workers who reported that they felt encouraged to conduct MPDR or thought that MPDR affected how they provided maternal and new born care than those who did not. In addition, there was no difference in participation in MPDR among health workers who complained that MPDR increased their workload, or inconvenienced them than those who did not. However, health workers perception that MPDR improved maternal and new care were more likely to have participated in MPDR meeting (*p* < 0.04) Table 5.

### Qualitative findings

Findings from qualitative data showed that major health work force factors that influenced the conduct of MPDR complemented the quantitative data. These were grouped into: Functionality of maternal and perinatal review committees, and service delivery and health work force challenges to conduct of MPDR.

Service delivery and health work force challenges to conduct of MPDR included: recommendations are not being implemented, inadequate support supervision, difficulty in accessing information, heavy workload, lack of system in which MPDR is conducted and lack of MPDR committee support. Health work force challenges included: the lack of training and motivation, the fear of litigation, the low number of staff and staff attitude towards conducting MPDR.

Suggested solutions to address the service delivery challenges included: implementation of MPDR recommendations, support supervision, building systems to facilitate MPDR reporting, schedule review meetings and formation of MPDR committees in health facilities without. Suggested solutions to address health workforce challenges included: continuous training all other health workers in MPDR, continuous medical education to update health worker skills, to address causes of maternal and perinatal deaths, recruitment of more staff, and designing strategies of retaining staff.

### Functionality and the perceived role of maternal and perinatal review committees

The district maternal and perinatal death review committee existed in Oyam district, and comprised of ten members. The Majority of the KIs reported that their role was to review MPDR reports and minutes from health facilities. The District MPDR committee was functional as shown by the minutes of meetings. Less than half of the KIs mentioned that their roles included follow-up on and implementation of recommendations, mobilization of resources, training of health workers and coordination of MPDR in the district. However, no one among the KIs reported that they had the role of ensuring that the maternal and perinatal deaths were notified or reviewed (Table 6).

Four of the seven health facilities had MPDR committees. However, most of the respondents did not know when the MPDR were started in their health facilities. The notification (within 24 h) and conduct (within 7 days) of MPDR were not done according to the Ministry of Health guidelines. Committees have been reviewing maternal and perinatal deaths monthly but even this has not been successful because of the heavy workload, competing activities making it difficult to have the review team sit together, and the many perinatal deaths making it impossible to review all of them.

Table 7 presents qualitative findings of service delivery and health work force challenges to conduct of MPDR and the proposed solutions by the KIs.

**Table 7:** Summary of reported service delivery challenges and proposed solutions

Service delivery challenges	No. of respondents
*Factors influencing identification of cases*	
Delay of mothers to reach the health facility	**
Only health facility deaths are reviewed	*
Difficult to collect information from the community	*
*Factors influencing review meeting*	
Inadequate support supervision	***
Non functionality of the existing MPDR committees	***
Heavy workload	***
Lack of copies of policy documents, guidelines, protocols	**
No MPDR committees	*
High number of perinatal deaths	*
*Factors influencing use of findings*	
Inadequate funding	**
Non implementation of recommendations	*
Late release of funds	*
No follow-ups by the district	*
Solutions to the service delivery challenges	
*Identification of cases*	
Training of Village health teams	***
Community sensitization on benefits of MPDR and need to seek health care services early	*
Early referral from the lower level units	*
*Review meetings*	
Provision of allowances during MPDR meetings	***
Formation of MPDR committees	**
Rescheduling of review meetings to less busy days	*
*Use of findings*	
District and MoH should implement review recommendations	***
Support supervision of health facilities	***
Ministry of health to fund MPDR activities for sustainability	*
Follow-up on health facilities to implement review recommendations	**
Health workforce challenges and proposed solutions in conducting MPDR	
Challenges	
Lack of training of MPDR committee members	***
Lack of allowances to motivate MPDR committee members	***
Lack of training of health workers on MPDR	***
Fear of litigation	**
Few health workers	*
Reluctance of MPDR committee members	*
Staff attrition	*
Proposed solutions	
Train members of MPDR committee	***
Train all health workers on MPDR	***
Conduct continuous medical education on MPDR	**
Recruit more staff	*
Design staff retention policy at district/health facility level	*

Notes: Data sourced from field: findings from KII;* Few respondents, **majority respondents, ***Most respondents

#### Service delivery challenges to conduct MPDR

The Majority of respondents reported that their main challenge to conducting MPDR was inadequate support supervision from the district and MOH. There was no particular support supervision for maternal and perinatal death reviews and it was integrated with other support supervisions that are conducted quarterly and often they only collect reports as shown below:

“*Support supervision by the district is almost not there at all apart from some members from the district health office coming to collect reports, and once in a while we get supervisors from the ministry of health”.* KI (In- charge HF)

Almost all respondents voiced the loss of morale to conduct MPDR when recommendations are not being implemented. The issue was that they keep discussing same problems in subsequent meetings because they are not implemented.

*“None implementation of recommendations by those above the health facility level demotivates MPDR members. You keep discussing the same recommendations and no actions are taken by the district”.* KI (In-charge HF)

Majority of respondents reported that the lack of funds made implementation of recommendations at community level particularly difficult especially if death was as a result of community delays that needed community level actions

*“Mothers always delay to come to the health facility and they end up losing their babies, we have always recommended that community sensitization be conducted, but we do not have funds to facilitate health workers to conduct community sensitizations”.*

In addition, respondents echoed a difficulty in collecting information from the community to complete the story to death as illustrated below:

*“It is very difficult to collect information from the community most especially when death was due to delay to seek service, they would want to blame it on health workers. They don’t want to admit that they delayed to take the woman to the health facility”.* KI (Chairperson MPDR Committee)

Heavy workload with fewer staff, and finding adequate time for staff to carry out review activities were among the principal barriers to institutionalizing reviews.

Lack of facilitation to the MPDR committees was reported by majority of the respondents. KIs elaborated that previously, Pathfinder International a global non-governmental organization gave them some incentives and when these stopped so did the morale to conduct reviews as shown below:

*Pathfinder used to give incentives to members and they got used to the allowance of 30,000/=, now calling members for a meeting knowing there is no allowance is a challenge since meetings last more than 2 h”.* KI (MPDR Committee member)

Respondents reported that meetings were always conducted during lunch hours and they lasted long. The lack of funds to provide members with lunch or a soft drink was very demotivating making it challenging to get members come for the meeting.

*“We trap the staff for the review meeting at lunch time when they are supposed to go for lunch but we can’t even offer drinks or lunch allowance to them”.* KI (Chairperson MPDR Committee).

Furthermore, respondents reported the lack of system in which MPDR is conducted. Health workers often have to use their personal resources for death notifications and to submit reports to the district or ministry of health as illustrated below:

*“We spend our personal money to call the DHO or MoH to notify them about the occurrence of maternal death. As for the review reports, they are submitted when someone is travelling to the district or MoH. There is no particular facilitation for submission of reports”* by KI (In-charge health facility (HF)).

### Health work force challenges *to conduct MPDR*

Almost all the KIs mentioned a lack of training of MPDR committee members. In addition, lack of training of other health workers who are not members of MPDR committees was another challenge. MPDR is an investigation of circumstances that surround maternal and perinatal death; and all health workers who are involved in provision of maternal and child health care need to know what MPDR is about in order to support its recommendations. Other challenges mentioned were fears of litigation of health workers by government because of the maternal death leading to reluctance of health workers to conduct MPDR, and staff attrition.

### Suggested solutions to address the service delivery challenges

Most frequently suggested solutions to the service delivery factors were implementation of MPDR committee recommendations. Respondents reported that their review recommendations were used for planning, budgeting, identification of training needs and filling the gaps in care. If recommendations are acted upon, then members realize how useful MPDR is and will be motivated to continue. One gave an example below, which motivated health workers to continue their work.

*“During our reviews we realized that the pressure of mothers who had died from eclampsia was not monitored because all the pressure machines were old and had broken down, so were decided to procure a pressure machine for maternity ward. Now mothers do not die from undetected high blood pressures”.* KI 9.

The respondents underscored the need for support supervision to the MPDR committees. With the minimal training observed, support supervision and mentorship was highly recommended.

*“Support supervision should be undertaken by the district. I suppose when they were making the budget, UNFPA catered for support supervision, if they did not, let them include that budget”.* KI (In-charge HF)

The respondents emphasized building systems to facilitate MPDR committees for reporting. They observed that other NGOs facilitated units they worked in and could not understand why UNFPA could not do the same. With low remuneration of health workers, having to shoulder costs of reporting was not acceptable as shown below:

*“UNFPA should facilitate communication like other NGO’s are doing, they can provide approximately 30,000 per month for communication to lower level units, district and MoH because this is costly for an individual”.* KI (Chairperson MPDR Committee)

Furthermore, health facilities should ensure that there is a schedule for the review meetings even within the heavy workload. The staff should be informed about the review scheduled days and audit meetings shifted to less busy days so as to plan to leave other work and attend.

Majority of the respondents suggested that formation of MPDR committees in health facilities without needed urgent attention. In addition, they felt a need to train village health teams to collect information on maternal and perinatal deaths from the community; and conduct community sensitization on MPDR benefits.

*“Village health team is a structure by ministry of health, if they were involved through training they would do the maternal and perinatal death reviews at community level and they report to their nearest health facility, they should be trained to capture simple information of the cases and report to the health facilities”* KI (district health team).

#### Suggested solutions to address health workforce challenges

Training members of the maternal and perinatal review committee was suggested by most of the KIs to enhance conduct of MPDR. Training was thought to be a motivation to the members to when they understand the implication of the MPDR.

*“The MPDR committee should be trained other than just using guess-work or using our medical knowledge. Training would be one of the motivation factors, besides staffs don’t understand the benefit of MPDR, they think ministry of health is just imposing the reviews to them”.* KI (In-charge HF).

Furthermore, the need to train all other health workers was emphasized since MPDR recommendations are implemented at different sections of the health facility and not only in maternity as suggested below:

*“Train all cadre of health workers, not only In-charges of health facilities and Midwives because the cause of death can be from out patients department, so all health workers should be trained as a collective effort”* stressed KI (Chairperson MPDR Committee).

The other suggested solutions were conducting continuous medical education to update health worker skills on how to address some of the causes of maternal and perinatal deaths. In addition, recruitment of more staff, and designing strategies of retaining staff most especially in the private-not-for profit health facilities was suggested.

## Discussion

About 71 % of the maternal deaths and only 33.3 % perinatal deaths that occurred from 2008 to 2011 were reviewed. This implies that not all maternal deaths are being reviewed as required by policy. While it is not in policy to review all perinatal deaths, reviewing only 33 % is too low and may not be informative to bring out all the issues that explain why newborns die. MPDR when done well leads to reduction of maternal and perinatal mortality. The neighbour to Oyam (Katakwi district), where all (100 %) maternal deaths were reviewed had a reduction of maternal mortality in the district by 20.4 % [12]. KIs reported that the perinatal deaths were very many and the review committees could not get enough time to review all of them. In addition, the health workers complained that the heavy workload made it difficult to review all the perinatal deaths. On the contrary, in Malawi the high maternal mortality was reported as a motivator to review the maternal deaths, though it could also be a demotivator if health workers do not notice any decline in the deaths following reviews [13].

The Ministry of Health of Uganda identifies the failure to respond to the recommendations from review of maternal and perinatal deaths as the main weakness in the implementation of the strategy. This has contributed to the reluctance of health facilities to conduct reviews [14]. In our study, four of the seven (4/7) health facilities had MPDR committees but only 2 were functional. All the HC III’s did not have functional MPDR committees despite having trained the health workers. This finding is similar to findings in Tanzania where 4/8 hospitals studied had maternal death review committees [15]. The absence of functional MPDR committees was linked to limited staff, limited training of MPDR committee members, lack of commitment of key staff and leadership. In addition, the inadequate financial support, irregular supportive supervision and lack of follow-up could have contributed to the non-functioning of the committees. These findings are also reported by other studies in other countries [15–18]. Absence of committees means reviews cannot be conducted and health facilities will not learn from the deaths to prevent further deaths. A study in South Africa reported that training committee members was key in institutionalization of reviews [19]. Lack of training of MPDR members and other health workers posed a big challenge to conduct of MPDR. The review committees were reluctant to play their roles since there was no clear guidance on the MPDR process. The need to train all health workers on MPDR process to reinforce their knowledge on the procedures and importance of MPDR in reducing maternal and perinatal death reviews was also supported by other studies [16, 20].

The MPDR recommendations should be communicated to all concerned stakeholders for action and remedy of the gaps identified. This study found that there was communication of the MPDR recommendations to the staff that provide maternal and perinatal care. However, communication between health facility levels and different role-players such as the district, community and MOH was not well established. In Senegal the health workers complained of lack of communication between the review committees and staff making it difficult to implement the recommendations [21]. This study, like other studies, found that implementation of review recommendations influenced the conduct of MPDR [13, 22]. In Malawi implementation of the review recommendations reduced the incidence of uterine rupture by 68 % [23]. Non-implementation of recommendations devalues the importance of MPDR, which emphasizes taking action to reduce maternal and perinatal morbidity and mortality. Studies reported discouragement of staff and failure to improve the quality of care when review recommendations were not implemented [13, 15, 20, 24].

A critical role of the district MPDR committee was support supervision. However, this support supervision was integrated into others activities and this gave little focus to MPDR. The health workers did not appreciate support supervision for MPDR activities within this integrated activity. There was very poor follow-up by the district; especially to ensure that maternal and perinatal deaths are reviewed by the health facilities. This poor follow-up could have contributed to low coverage of maternal and perinatal deaths reviewed in the district. A study in the Sub-Saharan African countries also found limited support supervision of the MPDR activities when they were integrated into general support supervision and suggested targeted supportive follow-up as key to institutionalization of MPDR [25].

Furthermore, the limited number of health workers hindered the conduct of MPDR. Other studies also found out that the shortage of staff, combined with other competing obligations made it difficult to bring health workers together to conduct a review [13]. Another factor that influenced conduct of MPDR was review committee motivation. Most of the KIs reported that lack of financial motivation for the MPDR committee members during meetings hindered the review process. The meetings were always conducted during lunch hours and lasted long hence the need for lunch either in terms of refreshment or money. Similar views were reported by other studies that motivation is important for continued conduct of MPDR [13, 16, 21, 26].

### Limitations of the study

This study had limitations of incomplete health records/registers or poorly kept records at some health centres. In addition, information bias by KI interviews was likely because they would want to “prove” that the review process was working well. However, they were able to share the sensitive issues like their boldness of saying allowances hinder reviews and recommending allowances to motivate MPDR committee members yet they know that the government prefers that these meetings be part of their routine work and therefore should not attract allowances. The discussion of not ordering drugs in time, not using partographs highlighted their ability to discuss the gaps in their own care of patients. In addition, correctly outlining the roles of MPDR committee members indicates that the other information given may be correct. The small sample size did not allow further analysis to assess the factors attributed to conducting MPDR. However, the qualitative component provided understanding of respondent’s perceptions and views on factors that influence conduct of maternal and perinatal death reviews.

## Conclusions

The proportion of maternal and perinatal deaths reviewed in Oyam is lower than expected. The pre-implementation phase of maternal and perinatal death review system was not effectively done in Oyam district. This would have ensured that health workers learn about MPDR and understand its benefits of improving quality of maternal and perinatal care and be encouraged to start its implementation. The lack of proper initiation of the process led to reluctance of health facility management and health workers to conduct maternal and perinatal death reviews. Conditions that appeared essential for the implementation and sustainability of maternal and perinatal death reviews were the presence and functionality of maternal and perinatal death review committee, training of MPDR members, having senior staff and administrators attending MPDR meetings. Communicating review recommendations to other staff members, feedback and supportive supervision from a higher level in the health system, and a sense of accountability and motivation was also critical. Lastly, there was a lack of continuity with the community, yet some of the causes of maternal and newborn mortality were emanating from the families and community.

Therefore, the district should functionalize its maternal and perinatal death review committee so as to provide overall coordination, ensure effective support supervision, and manage the maternal and perinatal death review as part of its routine surveillance programs. The district should ensure that maternal and perinatal review committees are trained and supported in all the health facilities. The health facilities should ensure that MPDRs are conducted every month for all health facility maternal and perinatal deaths and implement the recommendations to prevent further causes of death of mothers and newborns. The Ministry of Health and the district should ensure constant funding of MPDR activities. The district should consider training village health teams to conduct verbal autopsy in addition to the health facility based reviews to helps reconstruct the whole story surrounding the woman’s or newborn’s “road to death”.

## Abbreviations

HC, health centre; HF, health facility; KI, key informants; LRA, lord’s resistance army; MDGs, Millennium development goals; MOH, ministry of health; MPDR, Maternal Perinatal Death Review; NCMPDR, National Committee on Maternal Perinatal Death Review

## Additional file

Additional file 1: Questionnaire: *Factors influencing the conduct of maternal and perinatal death audits in Oyam district. (DOCX 135 kb)*
