# Supplementary material for: The conduct of maternal and perinatal death reviews in Oyam District, Uganda: a descriptive cross-sectional study
Source: BMC Womens Health. 2016 Jul 14;16:38. doi: 10.1186/s12905-016-0315-5 (PMC4944522; doi:10.1186/s12905-016-0315-5)
Supplement: Additional file 1: — Questionnaire: Factors influencing the conduct of maternal and perinatal death audits in Oyam district. (DOCX 135 kb) [file 12905_2016_315_MOESM1_ESM.docx]

***Factors influencing the conduct of maternal and perinatal death audits in Oyam district***

# Appendix I: Instruments for Data Collection

# Instrument I (A): Key informant interview guide for district health team and in-charges of health facilities

*Start after self introduction and getting the consent from the respondent.*

**Service delivery factors**

1. How was maternal and perinatal death audit institutionalised in Oyam district? *(Probe for information on: -* ***Who initiated*** *the maternal and perinatal death audit process?* ***When*** *was MPDA initiated?* ***What activities*** *were undertaken to institutionalise MPDA process (formation and training of MPDA committees, training of health workers on MPDA, distribution of policy documents, data collection tools, and information, education and communication materials)*
2. How is the district health team involved in the maternal and perinatal death audit process?

*Probe for more information on* ***advocacy*** *to key stakeholders,* ***training*** *of health workers,* ***coordination,*** *guidance,* ***supervision****,* ***analysis*** *of data,* ***documenting*** *findings,* ***mobilization*** *and community sensitization)*

1. Which development partners are supporting maternal and perinatal death audit process in Oyam district? (*Probe for which partners are supporting the district)*
2. Which kind of support do the development partners give to the district or health facilities? *(Probe for financial support, technical support, supervision, provision of supplies, recruitment of staff)*
3. What are the challenges to conducting maternal and perinatal deaths audits *(Probe for challenges)*
4. How should these challenges be addressed? *(Probe for solutions)*

**Health information factors**

1. What audit reports do you receive from the health facilities? (*Probe for maternal death audit reports and perinatal death audit reports, maternal death notification reports, quarterly MPDA reports)*
2. How do you disseminate the maternal and perinatal deaths audit reports? (*Probe for to whom the reports are disseminated to, how they are disseminated and how often they are disseminated)*
3. What actions have you taken following the maternal and perinatal death audit recommendations? (*Probe for actions taken)*
4. How are the analyzed results and recommendations of the audit committees connected to the district database? *(Probe for information about how the information on maternal and perinatal audits are included in the district database)*

# Instrument I (B): Key informant interview guide for Chairpersons of MPDA Committee

**Health workforce factors**

1. Existence of maternal and perinatal deaths audit committees

(*Probe for more information on* ***availability*** *of maternal/ perinatal mortality audit committees,* ***number*** *of maternal/ perinatal mortality audit committees,* ***composition*** *of maternal/ perinatal mortality audit committee)*

1. Experience of conducting maternal and perinatal death audit *(Probe for more information on* ***responsibilities****,* ***involvement*** *in the maternal/ perinatal death audit committees in this hospital,* ***for how long s/he has been involved*** *in the committee)*
2. How is the audit process conducted? *Probe for information on* ***who initiates the process*** *of auditing, Do you conduct* ***audit on all deaths*** *or only some? If only some,* ***how are the deaths selected*** *for discussion?* ***How soon*** *after the death is an audit conducted?* ***How frequently*** *are the audits conducted?* ***Who are involved*** *in the audit process (their professionalism and managerial positions)?* ***Who are the participants*** *in a typical case of auditing?* ***How do they reach consensus*** *on recommendation?* ***How long does an audit take*** *to complete?]*
3. What are the challenges you face in conducting maternal and perinatal deaths audits ***(****Probe for challenges faced; limited staff, limited time, not trained in conducting audits, fear of litigation, no MPDA forms, lack of supervision etc)*
4. What solutions do you propose for the challenges you have mentioned? *(Probe for the solutions)*

**Information management factors**

1. How is information Managed *(Probe for what materials are used for documenting the process e.g. case notes, antenatal cards, partograms, delivery records etc?* ***Availability*** *of maternal/perinatal death audit forms, maternal death notification forms, and quarterly MPDA report forms, Availability of policy documents, guidelines and management protocols,* ***Completeness*** *of the records,* ***Who analyses*** *the results of an audit,* ***How feedback*** *to the care provider is given and* ***record keeping*** *)*
2. How are the analyzed results and recommendations of the audit committees connected to the health facility database? *(Interviewer: Probe for information about how the information on maternal and perinatal audits are included in the health facility database)*
3. How are recommendations of the audit committee disseminated to the stakeholders? *(Probe for information if the audit information is also disseminated to the peer health care providers within the health facility and referring one, institutional administration, Ministry of Health, District Health officer as well as the community).*
4. How are the audit recommendations used by the health facility managers and health policy makers in planning and budgeting in order to achieve desired changes in the health facility? *(Probe for more information about the use of the recommendations in planning, budgeting in order to achieve desired changes in the hospital, examples of the changes that have ever taken place as a result of implemented recommendation of the audit committee since its establishment, reasons for failures).*
5. What challenges do you face in Maternal and perinatal death audit information management? *(Probe for the challenges)*
6. What suggestions do you have to improve the information system in order to bring improvements in maternal and child health care in the health facility? *(Probe for the solutions)*

***Factors influencing the conduct of maternal and perinatal death audits in Oyam district***

# Instrument II: A semi-structured questionnaire for interview of health workers working in maternity and children’s wards

***Instructions:*** *Circle the appropriate code or fill the responses of the interviewee in the spaces provided after the question.*

**Part I: Identification**

1. Questionnaire number____________________________
2. Name of the Health facility________________________
3. Health facility level 1=Hospital 2=HC IV 3=HC III
4. Ownership of the health facility 1=Government 2=NGO(PNFP)
5. Location of health facility 1=Oyam North HSD 2=Oyam South HSD
6. Health worker’s category 1= Doctor 2=Clinical Officer 3= Midwife 4= Nurse 5= Others (specify) _____________________________
7. Section of the health facility 1= Maternity 2= Children’s ward 3= Outpatient department

**Part II: Awareness**

1. Have you ever heard about maternal and perinatal death audit? 1 =YES 2= NO

If Yes, please mention the reasons for establishment for its establishment

i. _________________________________________________________________________

ii. _________________________________________________________________________

iii. _________________________________________________________________________

1. Do you have a maternal and perinatal death audit committee in this health facility? 1= YES 2= NO
2. Do you remember how maternal and perinatal death audit was introduced in this health facility? 1= YES 2= NO
3. If yes in Question above, explain _*_____________________________________________*
4. Do you know the main objective of maternal and perinatal death audit? 1= YES 2= NO
5. If yes what is it? _____________________________________________________________
6. Were you trained on maternal and perinatal death audits? 1=YES 2=NO
7. Are there core members of MPDA committee that you know? 1= YES 2= NO

If yes, mention a few of them (Carder/Titles not names)

1. ______________________________ 3. ___________________________

2_______________________________ 4. ___________________________

1. Have the objectives of maternal and perinatal death audit been communicated to all members of the staff working in maternity/children’s wards? 1= YES 2= NO 3= DON’T KNOW

**Part III: Attitude**

1. Do you feel encouraged to conduct MPDA 1=YES 2=NO
2. Do you think audit committees can affect how **people** conduct maternal and newborn care in the health facility? 1= YES 2= NO 3= SOMETIMES
3. Do you think audit committees can affect how **you** conduct maternal and newborn care in this health facility? 1= YES 2= NO 3= SOMETIMES
4. How do you feel about conducting maternal/perinatal death audit?

1.________________________________________________________________________________

2.________________________________________________________________________________

3.________________________________________________________________________________

4_________________________________________________________________________________

**Part IV: Perceptions**

*I am going to ask you some questions and then you tell me your level of agreement whether you strongly agree, agree, disagree or strongly disagree.*

| **N0.** | **QUESTION** | **RESPONSE**  4=Strongest agree, 3=Agree,  2=Disagree, 1=Strongly disagree*(Circle one response)* | | | |
| --- | --- | --- | --- | --- | --- |
|  | In your opinion, does conducting maternal/perinatal death audit inconvenience you? | 4 | 3 | 2 | 1 |
|  | In your opinion does conducting MPDA increase your workload | 4 | 3 | 2 | 1 |
|  | In your opinion, has maternal /perinatal death audit improved maternal and child health services in your health facility? | 4 | 3 | 2 | 1 |
|  | Does the district or ministry of health offer enough opportunities (capacity building, technical support, documentation) to strengthen your knowledge in conducting maternal/perinatal death audit? | 4 | 3 | 2 | 1 |
|  | Do you consider that the available maternal/perinatal death **audit forms** in this health facility are enough/ adequate? | 4 | 3 | 2 | 1 |
|  | Do you consider that the available policy guidelines for conducting maternal/perinatal death audit in this health facility enough/adequate? | 4 | 3 | 2 | 1 |

**Part V: Practices**

1. Have **YOU EVER** participated in maternal and perinatal death audit meeting? 1= YES 2= NO
2. Do the senior members of this health facility attend the MPDA meetings?

1= YES 2= NO 3= SOMETIMES

1. Are both maternal and perinatal deaths that occur in this health facility audited? 1= YES 2= NO
2. If NO which deaths are **NOT** audited by the MPDA committee in this health facility? 1= Maternal deaths 2= Perinatal deaths 3= Both maternal and perinatal deaths
3. Do you know any recommendation that has been provided by maternal/perinatal death audit committee in this health facility? 1= YES 2= NO
4. If Yes, Mention them

1. _________________________________________________________________________

2. _________________________________________________________________________

3. _________________________________________________________________________

1. Do you know of any actions that were taken in this health facility because of maternal/perinatal deaths audit committee recommendations? 1= YES 2= NO
2. If Yes, Mention them

1. _________________________________________________________________________

2. _________________________________________________________________________

3. _________________________________________________________________________

If No why?_____________________________________________________________________

1. Have you observed any improvement on how maternal and newborn care is been provided in this health facility as a result of audit committee recommendations? 1= YES 2= NO
2. Do you get feedback from the MPDA committees? 1=YES 2=NO
3. How could functions of these MPDA committees be improved? Explain ________________________________________________________________________________________________________________________________________________________________________________________________________________________________________________________________________________________________________________________________________________________________________________________________________________________________________________________________________________________________________________________________________________________________________________________________________________________________________________________________________________

__________________________________________________________________________________

***Factors influencing the conduct of maternal and perinatal death audits in Oyam district***

# Instrument III: Check list

**General Characteristics of health facility**

1. Name of health facility____________________________________________
2. Health facility level 1=Hospital 2=HC IV 3=HCIII
3. Ownership of the health facility 1=Gov’t 2=NGO(PNFP)
4. Location of health facility 1=Oyam North HSD 2=Oyam South HSD

**Service delivery factors**

1. Does the health facility conduct maternal and perinatal death audits 1=YES 2=NO
2. Existence of MPDA Committee 1=YES 2=NO
3. Functionality of MPDA committee (existence of minutes of audits) 1=YES 2=NO
4. Supervision by the District conducted 1=YES 2=NO
5. MPDA recommendations implemented 1=YES 2=NO

**Availability of policy documents, data collection tools and IEC materials**

*Instruction: Make sure you observe these tools/documents*

1. National policy guidelines and standards for Sexual and Reproductive Health and Rights 1= YES 2= NO
2. Roadmap for Accelerated Reduction of maternal and neonatal morbidity and mortality in Uganda 1= YES 2= NO
3. Management protocols and guidelines for Emergency obstetric care(EmOC) 1= YES 2= NO
4. Maternal and perinatal death review guidelines 1= YES 2= NO
5. Maternal death audit forms 1= YES 2= NO
6. Perinatal death audit forms 1= YES 2= NO
7. Maternal death notification forms 1= YES 2= NO
8. Quarterly Maternal/Perinatal death review forms 1= YES 2= NO

**Proportion of maternal and perinatal deaths audited**

1. Total number of deliveries between 2008 and 2011__________________________
2. Total number of maternal death between 2008 and 2011______________________
3. Total number of maternal death notified between 2008 and 2011_______________
4. Total number of maternal death audited between 2008 and 2011_______________
5. Total number of perinatal death between 2008 and 2011______________________
6. Total number of perinatal death notified between 2008 and 2011_______________
7. Total number of perinatal death audited between 2008 and 2011_______________
8. Completeness of maternal register (all sections filled) 1=YES 2=NO
9. Completeness of Children’s register (all sections filled) 1=YES 2=NO

**Availability of health Personnel**

|  | *Carder* | *Recommended staffing level* | *No. of staff available* | *Staffing Gap* | *Not applicable* |
| --- | --- | --- | --- | --- | --- |
|  | Medical Officers |  |  |  |  |
|  | Clinical Officers |  |  |  |  |
|  | Anesthetic Officers |  |  |  |  |
|  | Theatre Attendants |  |  |  |  |
|  | Registered Nurses |  |  |  |  |
|  | Registered Midwives |  |  |  |  |
|  | Enrolled Nurses |  |  |  |  |
|  | Enrolled Midwives |  |  |  |  |
|  | Nursing Assistants |  |  |  |  |
